# Identification of Absorbed Constituents in the Rabbit Plasma and Cerebrospinal Fluid after Intranasal Administration of Asari Radix et Rhizoma by HS-SPME-GC-MS and HPLC-APCI-IT-TOF-MS^n^

**DOI:** 10.3390/molecules19044857

**Published:** 2014-04-17

**Authors:** Chen Li, Feng Xu, De-Mei Xie, Yu Jing, Ming-Ying Shang, Guang-Xue Liu, Xuan Wang, Shao-Qing Cai

**Affiliations:** State Key Laboratory of Natural and Biomimetic Drugs, School of Pharmaceutical Sciences, Peking University Health Science Center, No.38, Xueyuan Road, Beijing 100191, China; E-Mails: clearfont@163.com (C.L.); xufeng_pharm@163.com (F.X.); xlzh99@163.com (D.-M.X.); jing_jy@126.com (Y.J.); myshang@bjmu.edu.cn (M.-Y.S.); guangxl@bjmu.edu.cn (G.-X.L.); xuanwang6818@bjmu.edu.cn (X.W.)

**Keywords:** nasal therapy, *Asarum heterotropoides* var. *mandshuricum*, intranasal administration, effective substance, cerebrospinal fluid, absorption, SPME, GC-MS, LC-MS, additive effect

## Abstract

Traditional Chinese Medicine (TCM) nasal therapy has been utilized to treat numerous diseases for over two millennia. It has many advantages compared with other routes. In this article, headspace-solid phase microextraction-gas chromatography-mass spectrometry and high performance liquid chromatography-atmospheric pressure chemical ionization-ion trap-time of flight-multistage mass spectrometry were applied for the first time to analyze the absorbed constituents in rabbit plasma and cerebrospinal fluid (CSF) after intranasal administration of Asari Radix et Rhizoma (AR). In total, 47 absorbed AR constituents including 14 monoterpenes, 10 phenylpropanoids, four benzene derivatives, two alkanes, nine *N*-alkylamides and eight lignans were tentatively identified in the rabbit plasma and CSF. Thirty-three absorbed constituents are found to have different bioactivities related to the pharmacological actions of AR through bibliography data retrieval. These indicated that many types of constituents of TCM can be absorbed at the nasal cavity into both rabbit blood and CSF. This is the first study to explore the absorption of AR, and comprehensively analyze the absorbed constituents after intranasal administration of TCM. These findings extend our understanding of the effective substances of AR, and inspire us to make a hypothesis on the mechanism of additive effect of multiple constituents of TCMs, which is very worthy of further investigation.

## 1. Introduction

Traditional Chinese Medicine (TCM) nasal therapy (*Biliao* in Chinese) is used to treat local or systemic diseases through intranasal administration using TCMs in the form of powders or extracts. It has a long history of well over a thousand years since first recorded in *Huangdi Neijing* in the Warring State Period (403–221 B.C.), and is widely recorded in Chinese medical classics. It can be used to treat numerous maladies including internal, surgical, gynecological, pediatric and otolaryngologic diseases in TCM [1]. Intranasal administration has many advantages such as rapid onset of action, improved pharmacological effect, low side effect and good treatment compliance compared with oral and parenteral routes [2].

Several clinical researches regarding TCM nasal therapy were reported [3], and there are a few studies concerning the pharmacokinetics of absorbed constituents after intranasal administration of TCM preparations or single compound isolated from TCMs [4,5,6]. However, these reports mainly focused on determination of the contents of one or two absorbed constituents, and there is no report about the comprehensive analysis of absorbed constituents after intranasal administration of TCM. Besides, the techniques applied in previous researches (e.g., HPLC) lacked enough sensitivity to detect the trace constituents absorbed in the biological samples such as cerebrospinal fluid (CSF).

Asari Radix et Rhizoma (AR), one of the most frequently used TCMs in TCM nasal therapy, is recorded in the Chinese Pharmacopeia as the dried roots and rhizomes of *Asarum heterotropoides* Fr. Schmidt *var. mandshuricum* (Maxim.) Kitag., *A. sieboldii* Miq. *var. seoulense* Nakai and *A. sieboldii* Miq. Its frequency of utilization ranks first among prescriptions to treat brain diseases [7] and second in the recipes to treat migraine [8]. It is clinically used to alleviate pain and rhinitis, mainly to treat headache. It has been reported to possess analgesic, anti-inflammatory, sedative, antispasmodic, anti-allergic, cardiovascular, antitussive, hypothermal and anticonvulsant effects [9,10]. However, there is no report regarding the analysis of absorbed constituents of AR after intranasal administration.

In this article, techniques with high sensitivity namely headspace-solid phase microextraction-gas chromatography-mass spectrometry (HS-SPME-GC-MS) and high performance liquid chromatography-atmospheric pressure chemical ionization-ion trap-time of flight-multistage mass spectrometry (HPLC-APCI-IT-TOF-MS^n^) are employed to analyze the absorbed constituents in the plasma and CSF of rabbits after intranasal administration of AR comprehensively. HS-SPME-GC-MS is introduced to identify the volatile absorbed constituents and HPLC-APCI-IT-TOF-MS^n^ is applied to identify the non-volatile absorbed constituents. Finally, bioactivities of the absorbed constituents related to the pharmacological effects of AR are summarized to give a better understanding of the effective substances of AR after intranasal administration. As far as we know, this is the first report to explore the absorption of AR, and comprehensively analyze the absorbed constituents after intranasal administration of a TCM.

## 2. Results and Discussion

### 2.1. Identification of Absorbed Constituents in Rabbit Plasma and CSF by HS-SPME-GC-MS

**Figure 1:**
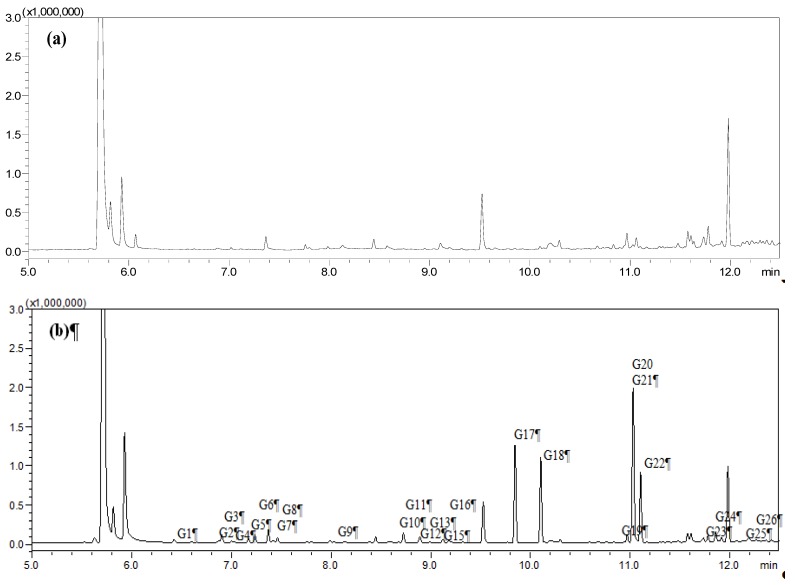
Total ion chromatograms of plasma obtained from (**a**) blank group and (**b**) Asari Radix et Rhizoma (AR) EtOAc extract group using HS-SPME-GC-MS.

**Figure 2:**
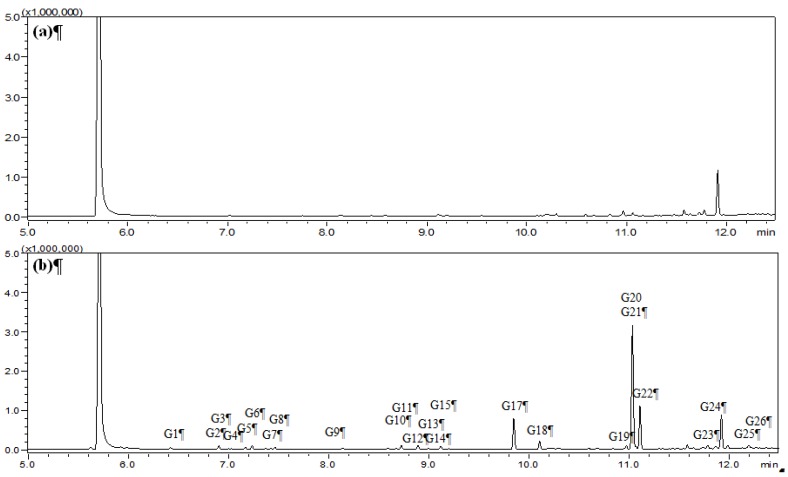
Total ion chromatograms of cerebrospinal fluid (CSF) obtained from (**a**) blank group and (**b**) AR EtOAc extract group using HS-SPME-GC-MS.

**Figure 3:**
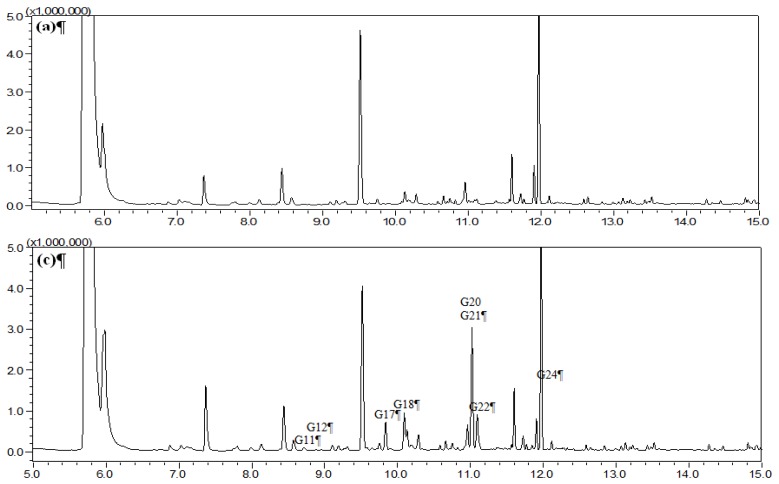
Total ion chromatograms of plasma obtained from (**a**) blank group and (**c**) Asari Radix et Rhizoma (AR) powder group using HS-SPME-GC-MS.

**Figure 4:**
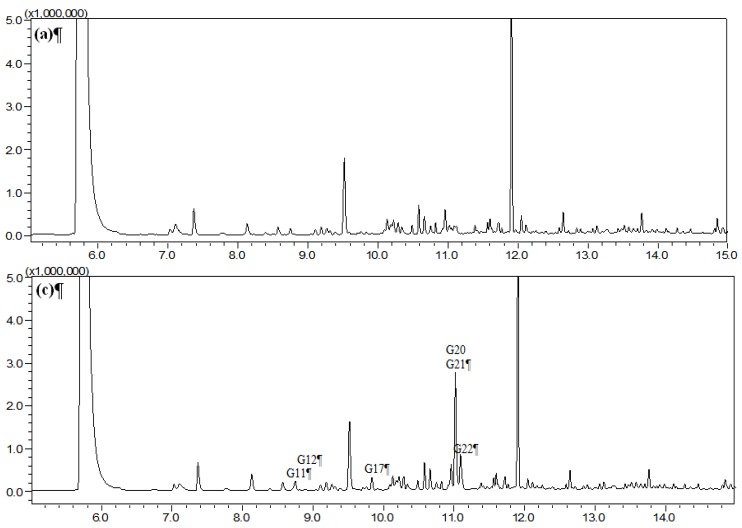
Total ion chromatograms of cerebrospinal fluid (CSF) obtained from (**a**) blank group and (**c**) Asari Radix et Rhizoma (AR) powder group using HS-SPME-GC-MS.

**Figure 5:**
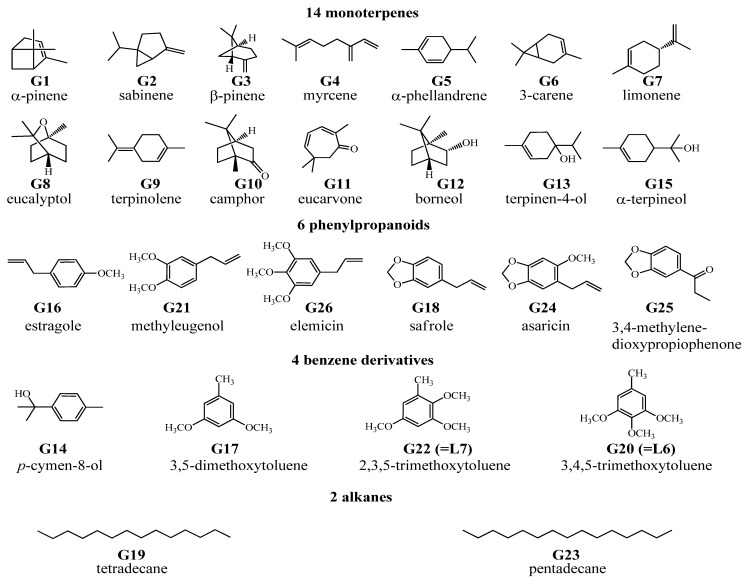
Chemical structures of 26 absorbed constituents identified in rabbit plasma and CSF from AR EtOAc extract group and the AR powder group identified by HS-SPME-GC-MS.

HS-SPME was chosen as the extraction mode because in this mode the SPME fiber can avoid the direct contact with biological samples, which can protect the fiber and prolong the fiber lifetime. Figure 1, Figure 2, Figure 3, and Figure 4 show the total ion chromatograms of the analyzed samples. In total, 26 absorbed constituents containing 14 monoterpenes (**G1**–**G13**, **G15**), six phenylpropanoids (**G16**, **G18**, **G21**, **G24**–**G26**), four benzene derivatives (**G14**, **G17**, **G20**, **G22**) and two alkanes (**G19**, **G23**) were identified in the plasma and CSF from the AR EtOAc extract group compared with the blank group. The results are summarized in Table 1 and Figure 5.

The AR EtOAc extract was reported to be the effective part of AR in analgesic activity [11], thus this extract was selected to study the absorbed constituents of AR after intranasal administration. Among the 26 absorbed constituents identified in the AR EtOAc extract group, 25 constituents were found in the plasma except *p*-cymen-8-ol, whereas 25 constituents were detected in the CSF aside from estragole.

Since AR is usually used in the form of powder in TCM nasal therapy clinically, the absorbed constituents after intranasal administration of AR powder were also studied and the absorbed constituents are supposed to be the effective substances of AR when it is used in nasal therapy. In the AR powder group, eight absorbed constituents (eucarvone, *l*-borneol, 3,5-dimethoxytoluene, safrole, 3,4,5-trimethoxytoluene, methyleugenol, 2,3,5-trimethoxytoluene and asaricin) were identified in the plasma and six of them (except safrole and asaricin) were detected in the CSF.

### 2.2. Identification of Absorbed Constituents in Rabbit Plasma and CSF by HPLC-APCI-IT-TOF-MS^n^

To reveal more constituents that could be absorbed into plasma and CSF, LCMS-IT-TOF was employed. Both APCI and ESI sources were tested in positive and negative ion modes. It was found that APCI gave stronger ion signals in positive ion mode. Hence APCI was selected as the ion source in the subsequent experiments monitored in the positive ion mode.

The fragmentation behaviors of four reference compounds (for their names see Section 2.2.1 below) in APCI-MS^n^ were studied at first in order to facilitate the structure characterization of the absorbed constituents. Afterwards, the absorbed constituents were identified or tentatively characterized by the following procedures: (1) compare the base peak chromatograms (BPCs) of the experimental groups with that of the blank group to find characteristic peaks of them; (2) confirm the existence of them by comparing their characteristic extracted ion chromatograms (EICs) among 3 groups; (3) predict their formulae by high resolution MS data obtained by IT-TOF; (4) elucidate their structures by high resolution IT-TOF MS^n^ data and by comparison with literature; (5) confirm their structures by comparison with the reference compounds as far as possible.

**Table 1:** The absorbed constituents in rabbit plasma and cerebrospinal fluid (CSF) from Asari Radix et Rhizoma (AR) EtOAc extract group (group **b**) and AR powder group (group **c**) identified by HS-SPME-GC-MS.

Compound No.	t_R_ (min)	RI ^b^ [12]	Constituents	Group b		Group c	
				Plasma	CSF	Plasma	CSF
G1	6.423	944	*α*-Pinene	+	+	−	−
G2	6.853	982	Sabinene	+	+	−	−
G3	6.902	988	*β*-Pinene	+	+	−	−
G4	7.000	993	Myrcene	+	+	−	−
G5	7.170	1012	*α*-Phellandrene	+	+	−	−
G6	7.236	1018	3-Carene	+	+	−	−
G7	7.421	1035	Limonene	+	+	−	−
G8	7.463	1040	Eucalyptol	+	+	−	−
G9	8.031	1096	Terpinolene	+	+	−	−
G10	8.675	1158	Camphor	+	+	−	−
G11 ^a^	8.724	1162	Eucarvone	+	+	+	+
G12 ^a^	8.890	1178	*l*-Borneol	+	+	+	+
G13	8.992	1188	Terpinen-4-ol	+	+	−	−
G14	9.035	1192	*p*-Cymen-8-ol	−	+	−	−
G15	9.118	1200	*α*-Terpineol	+	+	−	−
G16	9.179	1206	Estragole	+	−	−	−
G17 ^a^	9.847	1275	3,5-Dimethoxytoluene	+	+	+	+
G18 ^a^	10.105	1300	Safrole	+	+	+	−
G19	10.971	1400	Tetradecane	+	+	−	−
G20 ^a^, G21 ^a^	11.030	1408	3,4,5-Trimethoxy-toluene/methyleugenol	+	+	+	+
G22 ^a^	11.106	1417	2,3,5-Trimethoxytoluene	+	+	+	+
G23	11.783	1501	Pentadecane	+	+	−	−
G24	11.860	1510	Asaricin	+	+	+	−
G25	12.191	1550	3,4-Methylenedioxy-propiophenone	+	+	−	−
G26	12.268	1560	Elemicin	+	+	−	−
Sum				25	25	8	6

^a^ Confirmed by comparison with reference compounds; ^b^ retention index; + detected; − not detected.

**Figure 6:**
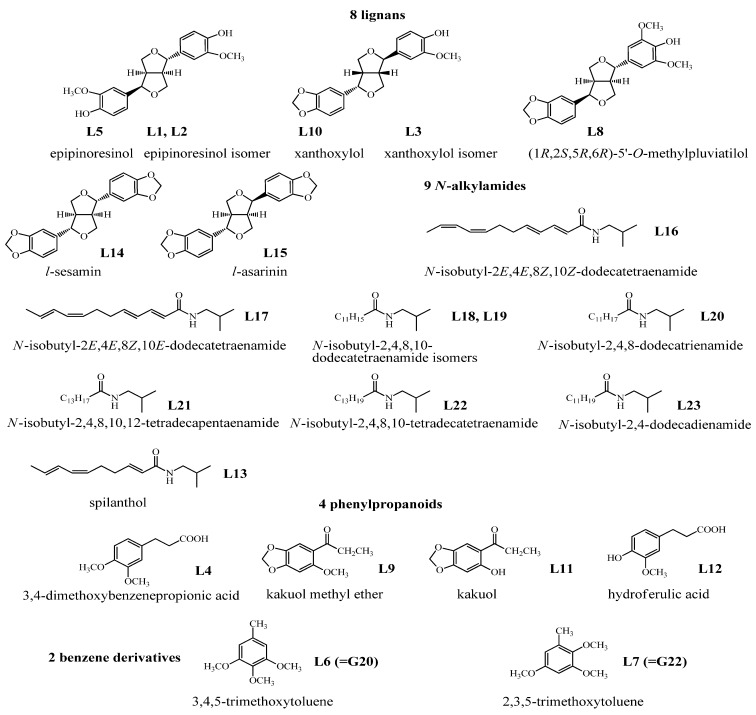
Chemical structures of 23 absorbed constituents identified in rabbit plasma from AR EtOAc extract group identified by HPLC-APCI-IT-TOF-MS^n^.

In total, 23 absorbed constituents including eight lignans (**L1**-**L3**, **L5**, **L8**, **L10**, **L14**, **L15**), nine *N*-alkylamides (**L13**, **L16**–**L23**), four phenylpropanoids (**L4**, **L9**, **L11**, **L12**) and two benzene derivatives (**L6**, **L7**) were identified in the plasma of the AR EtOAc extract group, but only five of them (**L6**, **L7**, **L15**–**L17**) were detected in the CSF. As for the AR powder group, only two compounds (**L16**, **L17**) were detected in the plasma and no compounds could be detected in the CSF. Among the above compounds, **L4**, **L8**, **L12**, **L13**, and **L20**–**L23** were the compounds which did not reported in *A. heterotropoides* var. * mandshuricum*. The results are summarized in Table 2 and Figure 6.

**Table 2:** The absorbed constituents in rabbit plasma and CSF from AR EtOAc extract group (group **b**) and AR powder group (group **c**) identified by HPLC-APCI-IT-TOF-MS^n^.

No.	t_R_ (min)	Meas. (Da)	Pred. (Da)	Err.	DBE	Formula	Identification results	Characteristic fragment ions	Group b		Group c	
				(ppm)					P	C	P	C
**L1**	11.486	359.1501	359.1489	3.34	10	C_20_H_22_O_6_	Epipinoresinol isomer	341.1380, 323.1279, 291.1026, 271.1010, 259.0748, 137.0618	+	−	−	−
**L2**	14.864	359.1489	359.1489	0	10	C_20_H_22_O_6_	Epipinoresinol isomer	341.1373, 323.1263, 291.1021, 271.0964, 259.0781, 137.0620	+	−	−	−
**L3**	22.813	357.1325	357.1333	−2.24	11	C_20_H_20_O_6_	Xanthoxylol isomer	339.1218, 321.1155, 291.0999, 289.0903, 269.0786, 137.0464, 135.0417	+	−	−	−
**L4**	26.497	211.0955	211.0965	−4.74	5	C_11_H_14_O_4_	3,4-Dimethoxybenzenepropionic acid	193.0846, 178.0611, 165.0904, 161.0598, 133.0630	+	−	−	−
**L5 ^a^**	27.031	359.1489	359.1489	0	10	C_20_H_22_O_6_	Epipinoresinol	341.1372, 323.1263, 291.1021, 271.0946, 259.0781, 137.0620	+	−	−	−
**L6 ^a^**	31.577	183.1007	183.1016	−4.92	4	C_10_H_14_O_3_	3,4,5-Trimethoxytoluene	168.0772, 152.0825, 151.0765	+	+	−	−
**L7 ^a^**	35.188	183.1007	183.1016	−4.92	4	C_10_H_14_O_3_	2,3,5-Trimethoxytoluene	168.0772, 152.0825, 151.0723	+	+	−	−
**L8**	36.811	387.1428	387.1438	−2.58	11	C_21_H_22_O_7_	(1*R*,2*S*,5*R*,6*R*)-5´-*O*-Methylpluviatilol	369.1354, 351.1237, 339.1198, 319.0954, 299.0947, 167.0723, 135.0462	+	−	−	−
**L9 ^a^**	37.013	209.08	209.0808	−3.83	6	C_11_H_12_O_4_	Kakuol methyl ether	191.0710, 176.0459, 161.0614, 133.0621	+	−	−	−
**L10 ^a^**	38.435	357.1333	357.1333	0	11	C_20_H_20_O_6_	Xanthoxylol	339.1222, 321.1147, 291.1048, 289.0874, 269.0807, 137.0545, 135.0423	+	−	−	−
**L11 ^a^**	38.733	195.0644	195.0652	−4.1	6	C_10_H_10_O_4_	Kakuol	177.0522, 147.0418, 139.0386, 137.0179, 119.0478, 109.0261	+	−	−	−
**L12**	39.104	197.0804	197.0808	−2.03	5	C_10_H_12_O_4_	Hydroferulic acid	180.0751, 179.0720, 155.0732, 151.0756, 133.0620, 123.0894	+	−	−	−
**L13**	51.123	222.1842	222.1852	−4.5	4	C_14_H_23_N_O_	Spilanthol	167.1237, 166.1329, 152.0970, 149.0985, 123.1139, 121.1017, 81.0807	+	−	−	−
**L14**	51.317	355.1191	355.1176	4.22	12	C_20_H_18_O_6_	*l*-Sesamin	337.1068, 319.0966, 289.0837, 261.0917, 231.0791, 203.0856, 135.0418	+	−	−	−
**L15 ^a^**	54.163	355.1178	355.1176	0.56	12	C_20_H_18_O_6_	*l*-asarinin	337.1075, 319.0978, 289.0875, 261.0916, 231.0784, 203.0850, 135.0425	+	+	−	−
**L16**	56.775	248.2016	248.2009	2.82	5	C_16_H_25_NO	*N*-isobutyl-2*E*,4*E*,8*Z*,10*Z*-dodecatetraenamide	192.1362, 175.1124, 167.1304, 166.1217, 149.1325, 147.1173, 142.1218, 121.1007, 107.0847	+	+	+	−
**L17**	57.218	248.2005	248.2009	−1.61	5	C_16_H_25_NO	*N*-isobutyl-2*E*,4*E*,8*Z*,10*E*-dodecatetraenamide	192.1362, 175.1124, 167.1289, 166.1232, 149.1325, 147.1174, 142.1206, 121.0999, 107.0852	+	+	+	−
**L18**	58.08	248.1997	248.2009	−4.83	5	C_16_H_25_NO	*N*-isobutyl-2,4,8,10-dodecatetraenamide isomer	192.1452, 175.1116, 167.1284, 166.1255, 149.1296, 147.1160, 142.1191, 121.1002, 107.0820	+	−	−	−
**L19**	61.053	248.2004	248.2009	−2.01	5	C_16_H_25_NO	*N*-isobutyl-2,4,8,10-dodecatetraenamide isomer	175.1135, 167.1339, 149.1333, 147.1138, 133.0672, 121.1017, 107.0813	+	−	−	−
**L20**	62.647	250.215	250.2165	−5.99	4	C_16_H_27_NO	*N*-isobutyl-2,4,8-dodecatrienamide	194.1625, 177.1164, 167.1302, 152.1070, 149.1398, 109.0863, 95.0789	+	−	−	−
**L21**	63.995	274.2164	274.2165	−0.36	6	C_18_H_27_NO	*N*-isobutyl-2,4,8,10,12-tetradecapentaenamide	201.1332, 175.1411, 173.1332	+	−	−	−
**L22**	66.168	276.2322	276.2322	0	5	C_18_H_29_NO	*N*-isobutyl-2,4,8,10-tetradecatetraenamide	220.1623, 203.1457, 177.1611, 175.1431, 167.1297, 135.1181, 133.0970	+	−	−	−
**L23**	66.563	252.2317	252.2322	−1.98	3	C_16_H_29_NO	*N*-isobutyl-2,4-dodecadienamide	196.1744, 179.1256, 154.1227, 95.0480	+	−	−	−
**Sum**									23	5	2	0

^a^ Confirmed by comparison with reference compounds; DBE, double bond equivalent; + detected; − not detected; P plasma; C cerebrospinal fluid.

#### 2.2.1. Fragmentation Behaviors of Four Reference Compounds in APCI-MS^n^

*Fragmentation behavior of* *l-asarinin (molecular formula: C_20_H_18_O_6_; exact mass: 354.1103)*. The quasi-molecular ion [M+H]^+^ of *l*-asarinin was observed at *m/z* 355.1178, and the base peak ion [M+H−H_2_O]^+^ at *m/z* 337.1045 was observed in MS spectrum. In MS^2^ spectrum, the precursor ion [M+H−H_2_O]^+^ at *m/z* 337.1045 (C_20_H_17_O_5_) fragmented into product ions at *m/z* 319.0955 (C_20_H_15_O_4_, [M+H−2H_2_O]^+^), *m/z* 289.0846 (C_19_H_13_O_3_, [M+H−2H_2_O−CH_2_O]^+^), *m/z* 261.0901 (C_18_H_13_O_2_, [M+H−2H_2_O−CH_2_O−CO]^+^), *m/z* 231.0803 (C_17_H_11_O, [M+H−2H_2_O−CH_2_O−CO−CH_2_O]^+^), *m/z* 203.0885 (C_16_H_11_, [M+H−2H_2_O−CH_2_O−CO−CH_2_O−CO]^+^), and *m/z* 135.0434 [C_8_H_7_O_2_]^+^. The ion at *m/z* 135.0434 [C_8_H_7_O_2_]^+^ is a very important characteristic fragment ion for identifying the structure of the aryl group in furofuran lignans, which indicated that the aryl group was 3,4-methylenedioxyphenyl [13,14]. In the MS^3^ spectrum, the precursor ion [M+H−2H_2_O−CH_2_O]^+^ at *m/z* 289.0846 further fragmented into product ions at *m/z* 261.0929 (C_18_H_13_O_2_, [M+H−2H_2_O−CH_2_O−CO]^+^), *m/z* 231.0795 (C_17_H_11_O, [M+H−2H_2_O−CH_2_O−CO−CH_2_O]^+^), and *m/z* 203.0864 (C_16_H_11_, [M+H−2H_2_O−CH_2_O−CO−CH_2_O−CO]^+^).

*Fragmentation behavior of* *epipinoresinol (molecular formula: C_20_H_22_O_6_; exact mass: 358.1416)*. In MS spectrum, the quasi-molecular ion [M+H]^+^ of epipinoresinol was observed at *m/z* 359.1444, and the base peak ion [M+H−H_2_O]^+^ at *m/z* 341.1363 (C_20_H_21_O_5_) was observed. In the MS^2^ spectrum, the precursor ion [M+H−H_2_O]^+^ at *m/z* 341.1363 (C_20_H_21_O_5_) fragmented into product ions at *m/z* 323.1258 (C_20_H_19_O_4_, [M+H−2H_2_O]^+^), *m/z* 291.0994 (C_19_H_15_O_3_, [M+H−2H_2_O−CH_3_OH]^+^), *m/z* 263.1042 (C_18_H_15_O_2_, [M+H−2H_2_O−CH_3_OH−CO]^+^), *m/z* 259.0734 (C_18_H_11_O_2_, [M+H−2H_2_O−2CH_3_OH]^+^), and *m/z* 137.0596 ([C_8_H_9_O_2_]^+^). The successive losses of two CH_3_OH from the ion *m/z* 323.1258 indicated that there were two *o*-methoxy hydroxyphenyls in the structure, and the characteristic ion at *m/z* 137.0596 ([C_8_H_9_O_2_]^+^) further confirmed this. In MS^3^ spectrum, the precursor ion [M+H−2H_2_O]^+^ at *m/z* 323.1186 generated product ions at *m/z* 291.1024 [M+H−2H_2_O−CH_3_OH]^+^ and *m/z* 263.1052 [M+H−2H_2_O−CH_3_OH−CO]^+^; the precursor ion at *m/z* 291.0094 (C_19_H_15_O_3_) gave product ions at *m/z* 276.0793 (C_18_H_12_O_3_) by losing a methyl radical, which also indicated that there was a methoxyl group in the molecule.

*Fragmentation behavior of kakuol (molecular formula: C_10_H_10_O_4_; exact mass: 194.0579)*. In MS spectrum, kakuol showed quasi-molecular ion [M+H]^+^ at *m/z* 195.0648. The quasi-molecular ion [M+H]^+^ gave fragment ions at *m/z* 177.0522 (C_10_H_9_O_3_, [M+H−H_2_O]^+^), *m/z* 166.0109 (C_8_H_6_O_4_, [M+H−C_2_H_5_]^+^), *m/z* 147.0418 (C_9_H_7_O_2_, [M+H−H_2_O−CH_2_O]^+^), *m/z* 139.0386 (C_7_H_7_O_3_, [M+H−C_3_H_4_O]^+^), *m/z* 137.0179 (C_7_H_5_O_3_, [M+H−C_3_H_6_O]^+^) , *m/z* 119.0478 (C_8_H_7_O, [M+H−H_2_O−CH_2_O−CO]^+^), *m/z* 109.0261(C_6_H_5_O_2_, [M+H−C_3_H_4_O−CH_2_O]^+^ or [M+H−C_3_H_6_O−CO]^+^), and *m/z* 95.0509 (C_6_H_7_O) in MS^2^ spectrum.

*Fragmentation behavior of* *kakuol methyl ether (molecular formula: C_11_H_12_O_4_; exact mass: 208.0736)*. It showed [M+H]^+^ at *m/z* 209.0801 in MS spectrum, and the characteristic product ions at *m/z* 191.0717 (C_11_H_11_O_3_, [M+H−H_2_O]^+^), *m/z* 176.0460 (C_10_H_8_O_3_, [M+H−H_2_O−CH_3_]^+^), *m/z* 161.0673 (C_10_H_9_O_2_, [M+H−H_2_O−CH_2_O]^+^), 133.0614 (C_9_H_9_O, [M+H−H_2_O−CH_2_O−CO]^+^) were observed in MS^2^ spectrum of [M+H]^+^. The precursor ion [M+H−H_2_O−CH_3_]^+^ at *m/z* 176.0460 gave a product ion at *m/z* 147.0423 (C_9_H_7_O_2_, [M+H−H_2_O−CH_3_−CHO]^+^) in MS^3^ spectrum.

#### 2.2.2. Characterization of Eight Absorbed Lignans of AR by HPLC-APCI-IT-TOF-MS^n^

Most lignans in AR belong to furofuran type. The structure skeleton, common substituent groups and diagnostic fragment ions of this type lignan are summarized in Table 3 according to our research and literature data [13,14,15,16].

**Table 3:** The structure skeleton, common substituent groups and diagnostic positive APCI-MS^n^ fragment ions of furofuran lignans.

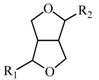				
R_1_	R_2_	R_1_ diagnostic ion	R_2_ diagnostic ion	MS base peak ion [M+H−H_2_O]^+^
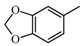	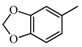	135.04 (C_8_H_7_O_2_)	135.04 (C_8_H_7_O_2_)	337.10 (C_20_H_17_O_5_)
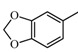	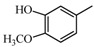	135.04 (C_8_H_7_O_2_)	137.06 (C_8_H_9_O_2_)	339.12 (C_20_H_19_O_5_)
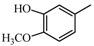	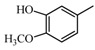	137.06 (C_8_H_9_O_2_)	137.06 (C_8_H_9_O_2_)	341.14 (C_20_H_21_O_5_)
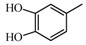	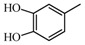	123.04 (C_7_H_7_O_2_)	123.04 (C_7_H_7_O_2_)	313.10 (C_18_H_17_O_5_)
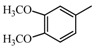	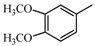	151.07 (C_9_H_11_O_2_)	151.07 (C_9_H_11_O_2_)	369.17 (C_22_H_25_O_5_)
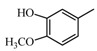	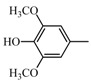	137.06 (C_8_H_9_O_2_)	167.07 (C_9_H_11_O_3_)	371.15 (C_21_H_23_O_6_)
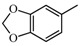	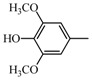	135.04 (C_8_H_7_O_2_)	167.07 (C_9_H_11_O_3_)	369.13 (C_21_H_21_O_6_)
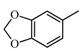	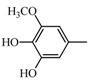	135.04 (C_8_H_7_O_2_)	153.05 (C_8_H_9_O_3_)	355.12 (C_20_H_19_O_6_)

*Identification of L1*, *L2*, *L5.* All of them showed [M+H]^+^ at *m/z* 359.14, and the base peak ion in MS was at *m/z* 341.14, indicating that their molecular formulae were C_20_H_22_O_6_. In MS^2^ spectra of them, the diagnostic ions at *m/z* 291.10 (C_19_H_15_O_3_, [M+H−2H_2_O−CH_3_OH]^+^), *m/z* 259.07 (C_18_H_11_O_2_, [M+H−2H_2_O−2CH_3_OH]^+^), and *m/z* 137.06 (C_8_H_9_O_2_) indicated that two aryl groups in **L1**, **L2** and **L5** were *o*-methoxy hydroxyphenyl. Further, **L5** was confirmed to be epipinoresinol by comparison with the reference compound, and **L1** and **L2** were tentatively identified as epipinoresinol isomers.

*Identification of L3 and L10*. The base peak ions of them were *m/z* 339.12 in MS, and the diagnostic ions of two aryl groups were observed at *m/z* 135.04 (C_8_H_7_O_2_) and *m/z* 137.06 (C_8_H_9_O_2_), accompanied with the ion at *m/z* 289.09 [M+H−2H_2_O−CH_3_OH]^+^ in MS^2^. This indicated that one aryl was *o*-methoxy hydroxyphenyl and the other was 3,4-methylenedioxyphenyl. Accordingly, **L10** was identified as xanthoxylol by comparison with the reference compound, and **L3** was tentatively identified as a xanthoxylol isomer.

*Identification of L8*. It showed [M+H]^+^ at *m/z* 387.14, and the base peak ion in MS was at *m/z* 369.13, indicating that its molecular formula was C_21_H_22_O_7_. The diagnostic ions of two aryl groups were observed at *m/z* 135.04 (C_8_H_7_O_2_) and *m/z* 167.07 (C_9_H_11_O_3_), accompanied with the ion at *m/z* 319.10 [M+H−2H_2_O−CH_3_OH]^+^ in MS^2^. This indicated that one aryl was *o*-methoxy dihydroxyphenyl and the other was 3,4-methylenedioxyphenyl. Therefore, **L8** was tentatively identified as (1*R*,2*S*,5*R*,6*R*)-5´-*O*-methylpluviatilol [17], which was isolated from *Asarum sieboldii.*

*Identification of L14 and L15*. **L15** was confirmed to be *l-*asarinin by comparison with reference compound. **L14** was an isomer of **L15**, which showed [M+H]^+^ at *m/z* 355.12 and the base peak ion [M+H−H_2_O]^+^ at *m/z* 337.10 in MS. The MS^2^ of **L15** showed characteristic product ions at *m/z* 289.08 (C_19_H_13_O_3_, [M+H−2H_2_O−CH_2_O]^+^) and *m/z* 135.0434 [C_8_H_7_O_2_]^+^, which was consistent with that of *l-*sesamin [15], so **L15** was tentatively identified as *l-*sesamin.

#### 2.2.3. Characterization of Nine Absorbed N-alkylamides of AR by HPLC-APCI-IT-TOF-MS^n^

The nine absorbed constituents (**L13**, **L16**–**L23**) were identified as *N*-isobutylamides, because of their characteristic neutral losses of 56 Da (C_4_H_8_), 73 Da (C_4_H_11_N), 99 Da (C_5_H_9_NO) and 101 Da (C_5_H_11_NO) from *N*-isobutylamide were observed in their MS^n^ spectra [18,19,20,21].

*Identification of L16-L19*. All of them showed [M+H]^+^ at *m/z* 248.20, which indicated that their molecular formulae were C_16_H_25_NO. The fragment ions at *m/z* 192.14 (C_12_H_18_NO, [M+H−C_4_H_8_]^+^), *m/z* 175.11 (C_12_H_15_O, [M+H−C_4_H_11_N]^+^), *m/z* 167.13 (C_10_H_17_NO, [M+H−C_6_H_9_]^+^), *m/z* 166.12 (C_10_H_16_NO, [M+H−C_6_H_10_]^+^), *m/z* 149.13 (C_11_H_17_, [M+H−C_5_H_9_NO]^+^), *m/z* 147.12 (C_11_H_15_, [M+H−C_5_H_11_NO]^+^), *m/z* 133.07 (C_9_H_9_O, [M+H−C_4_H_11_N−C_3_H_6_]^+^), *m/z* 121.10 (C_9_H_13_, [M+H−C_7_H_13_NO]^+^), and *m/z* 107.08 (C_8_H_11_, [M+H−C_7_H_13_NO]^+^) were observed in their MS^2^ spectra, which indicated that they had the degree of unsaturation of 5 and an alkyl chain of 12 carbons, and they were *N*-isobutyl-2,4,8,10-dodecatetraenamides [19]. Their UV spectra exhibited λ_max_ at 259 nm, which further confirmed that they were 2,4-diene alkamides. Besides, the peak areas of **L16** and **L17** were bigger than those of **L18** and **L19**, indicating that **L16** and **L17** were more abundant than **L18** and **L19**. Because *N*-isobutyl-2*E*,4*E*,8*Z*,10*E*-dodecatetraenamide and *N*-isobutyl-2*E*,4*E*,8*Z*,10*Z*-dodecatetraenamide were isolated from Asari Radix et Rhizoma [22], and the 2*E*,4*E*,8*Z*,10*Z*-isomer elute before 2*E*,4*E*,8*Z*,10*E*-isomer in HPLC [20], **L16** was tentatively identified as *N*-isobutyl-2*E*,4*E*,8*Z*,10*Z*-dodecatetraenamide and **L17** as *N*-isobutyl-2*E*,4*E*,8*Z*,10*E*-dodecatetraenamide. Besides, **L18** and **L19** were tentatively identified as *N*-isobutyl-2,4,8,10-dodecatetraenamide isomers.

*Identification of L20*. It showed [M+H]^+^ at *m/z* 250.2150, indicating the molecular formula of C_16_H_27_NO. Its UV spectrum exhibited λ_max_ at 262 nm, which indicated that **L20** was a 2,4-diene alkamide. The fragment ions at *m/z* 194.1625 (C_12_H_20_NO, [M+H−C_4_H_8_]^+^), *m/z* 177.1164 (C_12_H_17_O, [M+H−C_4_H_11_N]^+^), *m/z* 167.1302 (C_10_H_17_NO, [M+H−C_6_H_11_]^+^), *m/z* 152.1070 (C_9_H_14_NO, [M+H−C_7_H_14_]^+^), and *m/z* 149.1398 (C_11_H_17_, [M+H−C_5_H_11_NO]^+^) in MS^2^ of **L20** indicated that it had the degree of unsaturation of 4 and an alkyl chain of 12 carbons, and it was *N*-isobutyl-dodecatrienamide. By comparison with the literature [20], **L20** was tentatively identified as *N*-isobutyl-2,4,8-dodecatrienamide.

*Identification of L21*. It showed [M+H]^+^ at *m/z* 274.2164, indicating the molecular formula of C_18_H_27_NO. The UV spectrum of **L21** exhibited λ_max_ at 263 nm, which implied that it was a 2,4-diene alkamide. The characteristic fragment ions at *m/z* 201.1332 (C_14_H_17_O, [M+H−C_4_H_11_N]^+^), *m/z* 175.1411 (C_13_H_19_, [M+H−C_5_H_9_NO]^+^), and *m/z* 173.1332 (C_13_H_17_, [M+H−C_5_H_11_NO]^+^) in MS^2^ spectra of **L21** indicated that it had the degree of unsaturation of 6 and an alkyl chain of 14 carbons, and it was *N*-isobutyl-tetradecapentaenamide. Therefore, **L21** was tentatively identified as *N*-isobutyl-2,4,8,10,12-tetradecapentaenamide.

*Identification of L22*. It showed [M+H]^+^ at *m/z* 276.2322, which indicated that its molecular formula was C_18_H_29_NO. The UV spectrum of **L22** exhibited λ_max_ at 262 nm, which indicated that it was a 2,4-diene alkamide. The characteristic fragment ions at *m/z* 220.1623 (C_14_H_22_NO, [M+H−C_4_H_8_]^+^), *m/z* 203.1457 (C_14_H_19_O, [M+H−C_4_H_11_N]^+^), *m/z* 177.1611 (C_13_H_21_, [M+H−C_5_H_9_NO]^+^), *m/z* 175.1431 (C_13_H_19_, [M+H−C_5_H_11_NO]^+^), *m/z* 167.1297 (C_10_H_17_NO, [M+H−C_8_H_13_]^+^), *m/z* 135.1181(C_10_H_15_), and *m/z* 133.0970 (C_10_H_13_) in MS^2^ spectra of **L22** indicated that it had the degree of unsaturation of 5 and an alkyl chain of 14 carbons, and it was *N*-isobutyl-tetradecatetraenamide. Accordingly, **L22** was tentatively identified as *N*-isobutyl-2,4,8,10-tetradecapentaenamide.

*Identification of L23*. It showed [M+H]^+^ at *m/z* 252.2317, which indicated that its molecular formula was C_16_H_29_NO. The UV spectrum of **L23** exhibited λ_max_ at 262 nm, which indicated that it was a 2,4-diene alkamide. The characteristic fragment ions at *m/z* 196.1744 (C_12_H_22_NO, [M+H−C_4_H_8_]^+^), *m/z* 179.1256 (C_12_H_19_O, [M+H−C_4_H_11_N]^+^), *m/z* 154.1227 (C_9_H_16_NO, [M+H−C_7_H_14_]^+^), and *m/z* 95.0480 (C_6_H_7_O) in MS^2^ spectra of **L23** indicated that it had the degree of unsaturation of 3 and an alkyl chain of 12 carbons, and it was *N*-isobutyl-2,4-dodecaenamide. Accordingly, **L23** was tentatively identified as *N*-isobutyl-2,4-dodecaenamide.

*Identification of L13*. It showed [M+H]^+^ at *m/z* 222.1842, which indicated that its molecular formula was C_14_H_23_NO. The characteristic fragment ions at *m/z* 167.1237 (C_10_H_17_NO, [M+H−C_4_H_7_]^+^), *m/z* 166.1329 (C_10_H_16_NO, [M+H−C_4_H_8_]^+^), *m/z* 152.0970 (C_9_H_14_NO, [M+H−C_5_H_10_]^+^), *m/z* 149.0985 (C_10_H_13_O, [M+H−C_4_H_11_N]^+^), and *m/z* 81.0807(C_6_H_9_, [M+H−C_8_H_15_NO]^+^) in MS^2^ spectra of **L13** indicated that it had the degree of unsaturation of 4 and an alkyl chain of 10 carbons. According to literature [18], **L13** was tentatively identified as spilanthol, *i.e.*, *N*-isobutyl-2*E*,6*Z*,8*E*-decatrienamide, which was isolated from the roots and rhizomes of *Asarum longerhizomatosum* [23].

#### 2.2.4. Characterization of Four Absorbed Phenylpropanoids of AR by HPLC-APCI-IT-TOF-MS^n^

*Identification of L9 and L11*. **L9** and **L11** were confirmed to be kakuol methyl ether and kakuol by comparing their retention times and MS^n^ data with those of the reference compounds.

**Figure 7:**
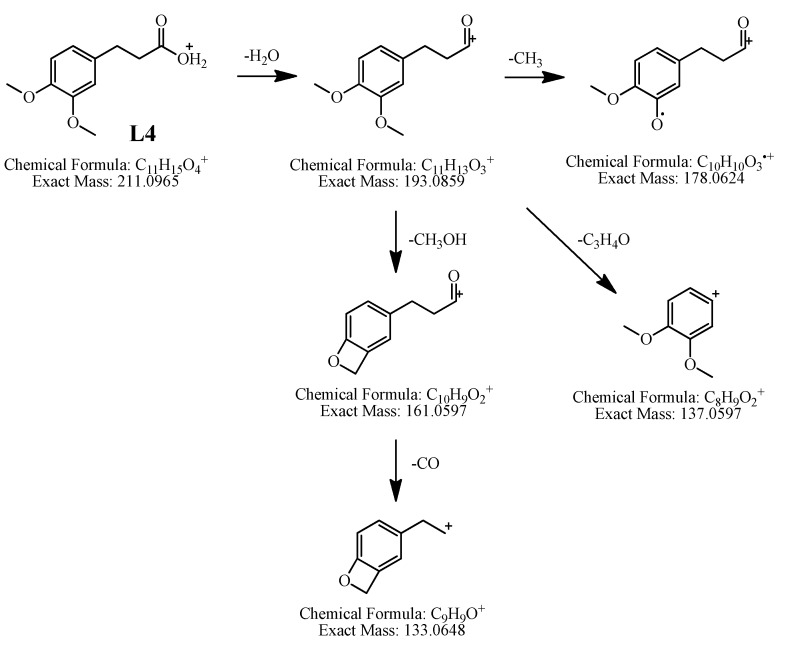
Proposed fragmentation pathways of 3,4-dimethoxybenzenepropionic acid (**L4**) in positive ion APCI-MS.

*Identification of L4*. It showed [M+H]^+^ at *m/z* 211.0955, indicating the molecular formula of C_11_H_14_O_4_. The precursor ion *m/z* 211.0955 (C_11_H_15_O_4_, [M+H]^+^) gave fragment ions at *m/z* 193.0846 (C_11_H_13_O_3_, [M+H−H_2_O]^+^), *m/z* 178.0611 (C_10_H_10_O_3_, [M+H−H_2_O−CH_3_]^+^), *m/z* 161.0598 (C_10_H_9_O_2_, [M+H−H_2_O−CH_3_OH]^+^), *m/z* 137.0541 (C_8_H_9_O_2_, [M+H−H_2_O−C_3_H_4_O]^+^) and *m/z* 133.0630 (C_9_H_9_O, [M+H−H_2_O−CH_3_OH−CO]^+^). According to these characteristic ions, **L4** was tentatively identified as 3,4-dimethoxybenzenepropionic acid. The fragmentation pathways of **L4** are proposed in Figure 7.

**Figure 8:**
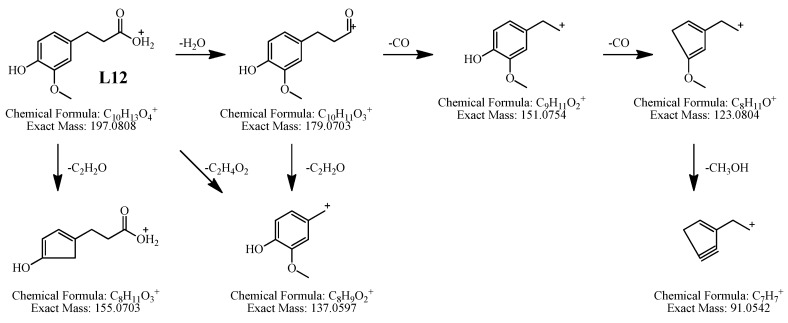
Proposed fragmentation pathways of hydroferulic acid (**L12**) in positive ion APCI-MS.

*Identification of L12*. It showed [M+H]^+^ at *m/z* 197.0804, indicating the molecular formula of C_10_H_12_O_4_. The precursor ion *m/z* 197.0804 (C_10_H_13_O_4_, [M+H]^+^) gave fragment ions at *m/z* 179.0720 (C_10_H_11_O_3_, [M+H−H_2_O]^+^), *m/z* 155.0732 (C_8_H_11_O_3_, [M+H−C_2_H_2_O]^+^), *m/z* 151.0756 (C_9_H_11_O_2_, [M+H−H_2_O−CO]^+^), *m/z* 137.0575 (C_8_H_9_O_2_, [M+H−H_2_O−C_2_H_2_O]^+^), *m/z* 123.0894 (C_8_H_11_O, [M+H−H_2_O−CO−CO]^+^), and *m/z* 91.0513 (C_7_H_7_, [M+H−H_2_O−CO−CO−CH_3_OH]^+^). According to these characteristic ions, **L12** was tentatively identified as hydroferulic acid. The fragmentation pathways of **L12** are proposed in Figure 8.

#### 2.2.5. Characterization of Two Absorbed Benzene Derivatives of AR by HPLC-APCI-IT-TOF-MS^n^

*Identification of L6 and L7*. Both of them showed [M+H]^+^ at *m/z* 183.10, indicating that their molecular formulae were C_10_H_14_O_3_. The fragment ions at *m/z* 168.07 (C_9_H_12_O_3_, [M+H−CH_3_]^+^), *m/z* 152.08 (C_9_H_12_O_2_, [M+H−CH_3_O]^+^), *m/z* 151.07 (C_9_H_11_O_2_, [M+H−CH_3_OH]^+^) were observed in their MS^2^ spectra. By comparison with reference compounds, **L6** and **L7** were unequivocally identified as 3,4,5-trimethoxytoluene and 2,3,5-trimethoxytoluene, respectively. These two compounds were also identified by HS-SPME-GC-MS technique in this study. **L6** was identical compound with **G20**, and **L7** was the same compound as **G22**.

### 2.3. Bioactivities of the Absorbed Constituents Related to the Pharmacological Effects of AR

Through HS-SPME-GC-MS and HPLC-APCI-IT-TOF-MS^n^, totally 47 absorbed constituents containing 14 monoterpenes, 10 phenylpropanoids, four benzene derivatives, two alkanes, nine *N*-alkylamides and eight lignans were identified in the rabbit plasma and CSF after intranasal administration of AR EtOAc extract. The numbers of identified absorbed constituents in different samples and by different methods are summarized in Table 4. It was found that these absorbed constituents were all the original constituents of AR and no metabolite was detected in the plasma and CSF. This may be because compounds avoid the gastrointestinal tract and first-pass metabolism through intranasal administration [24], indicating that these original constituents of AR might be the effective substances of AR when it is used in nasal therapy. Moreover, the molecular weight of these absorbed constituents were all less than 400 Da, and the number of the absorbed constituents having the molecular weight of less than 300 Da accounted for 83% of all the 47 absorbed constituents, which was consistent with the opinion that drugs with molecular weight lower than 300 Da are easy to be absorbed through intranasal administration [2]. AR powder comprised of lots of constituents with various polarities, and AR EtOAc extract was prepared from AR powder by extraction with EtOAc, a nonpolar solvent. Hence, AR EtOAc extract should be the less polar portion of AR powder. However, the absorbed constituents of AR powder are all found in the absorbed constituents of AR EtOAc extract. These findings suggested that the original constituents with small molecular weights and relatively low polarities might be the effective substances of AR when it is used in nasal therapy.

**Table 4:** The numbers of identified absorbed constituents in plasma and CSF of AR EtOAc extract group rabbits by HS-SPME-GC-MS and HPLC-APCI-IT-TOF-MS^n^.

	GC-MS	LC-MS	Same Constituents Identified by GC-MS and LC-MS	Total No.
Plasma	25	23	2 (**L6** = **G20**, **L7** = **G22**)	46
CSF	25	5	2 (**L6** = G20, **L7** = **G22**)	28
Same constituents in plasma and CSF	24 (**G1–G13, G15, G17–G26**)	5 (**L6, L7, L15–L17**)	2 (**L6** = G20, **L7** = **G22**)	27
Total No.	26	23	2	All: **47**

To find whether the identified absorbed constituents might be the effective substances of AR, their bioactivities related to the pharmacological effects of AR were summarized based on the overall literature retrieval. As shown in Table 5, 33 of 47 absorbed constituents were reported to exhibit diverse bioactivities associated with the pharmacological actions of AR (detail information are listed in Supplemental Materials Table S1). We can see that one absorbed constituent can have several bioactivities (Table S1; Table 5). Namely, methyleugenol has eight bioactivities, limonene and eucalyptol have seven bioactivities; three constituents (camphor, l-borneol and *α*-terpineol) have five bioactivities, six constituents have four bioactivities, three constituents have three bioactivities and six constituents have two bioactivities. These indicated that even one absorbed constituent could act on several targets simultaneously.

**Table 5:** Bioactivities of the absorbed constituents related to the pharmacological effects of Asari Radix et Rhizoma (AR).

Activity (number)	Structure Type (Number)	Constituents
Analgesic (14)	Monoterpene (9)	*α*-Pinene, *β*-Pinene, Myrcene, *α*-Phellandrene, Limonene, Eucalyptol, Camphor, *l*-Borneol, *α*-Terpineol
	Phenylpropanoid (3)	Estragole, methyleugenol, 3,4-Dimethoxybenzenepropionic acid
	*N*-alkylamide (2)	Spilanthol, *N*-Isobutyl-2*E*,4*E*,8*Z*,10*E*-dodecatetraenamide
Anti-inflammatory (27)	Monoterpene (12)	*α*-Pinene, Sabinene, *β*-Pinene, Myrcene, 3-Carene, Limonene, Eucalyptol, Camphor, Eucarvone, *l*-Borneol, Terpinen-4-ol, *α*-Terpineol
	Phenylpropanoid (5)	Estragole, Methyleugenol, Elemicin, Kakuol, Hydroferulic Acid
	Benzene derivative (1)	3,4,5-Trimethoxytoluene
	Lignan (4)	Epipinoresinol, (1*R*,2*S*,5*R*,6*R*)-5´-*O*-Methylpluviatilol, *l-*Sesamin, *l-*Asarinin
	*N*-Alkylamide (5)	Spilanthol, *N*-Isobutyl-2*E*,4*E*,8*Z*,10*Z*-dodecatetraenamide, *N*-Isobutyl-2*E*,4*E*,8*Z*,10*E*-dodecatetraenamide, *N*-Isobutyl-2,4,8-dodecatrienamide, *N*-Isobutyl-2,4-dodecadienamide
Sedative (5)	Monoterpene (3)	Myrcene, Limonene, Eucalyptol
	Phenylpropanoid (1)	Methyleugenol
	Benzene derivative (1)	3,5-Dimethoxytoluene
Anti-spasmodic (9)	Monoterpene (7)	Camphor, *l*-Borneol, *β*-Pinene, *α*-Phellandrene, Eucalyptol, Terpinolene, *α*-Terpineol
	Phenylpropanoid (2)	Methyleugenol, Estragole
Anti-allergic (8)	Monoterpene (2)	Limonene, *l*-Borneol
	Phenylpropanoid (2)	Methyleugenol, Elemicin
	Lignan (3)	Xanthoxylol, *l-*Sesamin, *l-*Asarinin
	*N*-alkylamide (1)	*N*-Isobutyl-2*E*,4*E*,8*Z*,10*E*-dodecatetraenamide
Cardiovascular (14)	Monoterpene (9)	*α*-Pinene, *β*-Pinene, Terpinen-4-ol, *α*-Terpineol, Eucalyptol, Camphor, *l-*Borneol, Limonene, Terpinolene
	Phenylpropanoid (5)	Methyleugenol, Elemicin, Kakuol, Hydroferulic Acid, Estragole
Antitussive (8)	Monoterpene (4)	α-Pinene , Camphor, Eucalyptol, Terpinen-4-ol
	Phenylpropanoid (1)	Kakuol
	*N*-alkylamide (3)	*N*-Isobutyl-2*E*,4*E*,8*Z*,10*Z*-dodecatetraenamide, *N*-Isobutyl-2*E*,4*E*,8*Z*,10*E* -dodecatetraenamide, *N*-Isobutyl-2,4,8,10,12-tetradecapentaenamide
Hypothermic (2)	Monoterpene (1)	Limonene
	Phenylpropanoid (1)	Methyleugenol
Anticonvulsant (5)	Monoterpene (4)	Myrcene, Limonene, Terpinen-4-ol, *α*-Terpineol
	Phenylpropanoid (1)	Methyleugenol

Furthermore, among them (Table 5), 14 absorbed constituents possess analgesic activities, 27 absorbed constituents induce anti-inflammatory effects, five absorbed constituents exhibit sedative prosperities, nine absorbed constituents have antispasmodic actions, eight absorbed constituents have anti-allergic effects, 14 absorbed constituents bring cardiovascular benefits, eight absorbed constituents act antitussive activities, two absorbed constituents show hypothermal actions and five absorbed constituents possess anticonvulsant effects. These indicated that many different constituents of AR might act at the same target where the additive concentrations of them could reach an effective level for disease treatment. This inspire us to make a hypothesis on the mechanism of additive effect of multiple constituents of TCMs, which is very worthy of further research in the future and the research evidence may provide us an explanation of why does a traditional Chinese drug still have effective actions although the concentrations of its constituents are generally very low in blood.

## 3. Experimental

### 3.1. Reagents and Materials

Asari Radix et Rhizoma (AR) was purchased from Anguo Herb Market (Anguo City, Hebei Province, China). The sample was identified as the roots and rhizomes of *Asarum heterotropoides* Fr. Schmidt var. *mandshuricum* (Maxim.) Kitag. by one of the authors, Professor Shao-Qing Cai, and its voucher specimen (No. 6650) was deposited in the Herbarium of Pharmacognosy, School of Pharmaceutical Sciences, Peking University (Beijing, China).

Methyleugenol (Lot: PH3YH-MG), eucarvone (Lot: F1101-DLFG) and *l*-borneol (Lot: EPH8L-QQ) were obtained from TCI (Tokyo, Japan), 3,4,5-trimethoxytoluene (Lot: 19923) from Aladdin Industrial Inc. (Shanghai, China), and 3,5-dimethoxytoluene (Lot: 10099004) from Alfa Aesar (Heysham, UK). Safrole, kakuol, kakuol methyl ether, epipinoresinol, xanthoxylol and *l*-asarinin were separated from Asari Radix et Rhizoma, and 2,3,5-trimethoxytoluene was synthesized by the authors. The structures were confirmed by MS and NMR, and their purities were over 98% (HPLC, area normalization method). *n*-Alkane mixture (C8-C20, Lot: 0001443129) was acquired from Fluka (Buchs, Switzerland) to calculate the retention indices (RI) of volatile constituents. Acetonitrile (HPLC grade) was purchased from Fisher (Fair Lawn, NJ, USA) and formic acid (HPLC grade) from Mreda Technology (Beijing, China). Sodium chloride was supplied by Sinopharm Chemical Reagent Co, Ltd (Beijing, China). Ethyl acetate (EtOAc) and methanol of analytical grade were obtained from Beijing Chemical Works (Beijing, China). Deionized water was prepared using a Milli-Q water purification system (Millipore, Billerica, MA, USA). The SPME fiber coated with 65 μm polydimethylsiloxane/divinylbenzene (PDMS/DVB) was purchased from Supelco (Bellefonte, PA, USA).

### 3.2. Preparation of AR EtOAc Extract

Fifty g of AR was ground and passed through a 40 mesh sieve (mesh size 0.425 mm) and then transferred into a 2000 mL flask containing 600 mL EtOAc. The flask was then sealed with a glass cap and placed in an ultrasonic bath at room temperature for 1 h for extraction. The extract was filtered and the filtrate was evaporated at 30 °C under vacuum using a Heidolph Laborota 4001 rotatory evaporator (Heidolph Instruments GmbH & Co., Schwabach, Germany) until no solvent was distilled, yielding 1.2 mL (1.26 g) oily AR EtOAc extract.

### 3.3. Animals and Sample Collection

The animal studies protocol was approved by the Biomedical Ethical Committee of Peking University (Approval No. LA2011-76). Male albino rabbits weighing 2.5 ± 0.1 kg were obtained from the Experimental Animal Center of Peking University Health Science Center (Beijing, China). 15 rabbits were randomly divided into three groups (five animals each): the AR EtOAc extract group (group **b**), the AR powder group (group **c**) and the blank group (group **a**).

The rabbits in the AR EtOAc extract group were administrated intranasally with a single dose of 50 µL AR EtOAc extract in each nostril using a microlitre syringe attached with polyethylene tube (2 mm in diameter). For intranasal administration of AR powder, the ground AR powder was passed through an 80 mesh sieve (mesh size 0.180 mm) according to the instruction for nasal powder of the Chinese Pharmacopeia. The powder was weighed and placed in a 1 mL pipette tip connected to a rubber suction bulb. The pipette tip was then inserted into the animal nostril and 0.05 g AR powder was blown into each nostril. The rabbits in the AR powder group received the dosing twice a day for 10 days (0.08 g/kg per day) before the blood and the CSF samples were collected. The rabbits in the blank group were administrated intranasally with deionized water. During the administration, all animals were held in a supine position and kept in this position for 1 min after drug administration.

Blood samples were withdrawn in heparinized vacuum glass tubes via cardiac puncture, and then the samples of each group were combined into one sample. The plasma was separated by centrifugation at 3000 rpm for 15 min. CSF samples were obtained by cisternal puncture using a 1 mL syringe with 30 gauge needle, and the samples of each group were combined into one sample. The samples of rabbits in the AR EtOAc extract group were collected at 15 min after administration, which was the time point that the highest nociception inhibition of stimulus was observed according to our previous pharmacological experiment. The samples of rabbits in the AR powder group were taken at the 10th day after administration. All samples were collected while the rabbits were under anesthesia, and the samples were pretreated and analyzed within 24 h.

### 3.4. Automated HS-SPME-GC-MS Analysis

A Shimadzu (Kyoto, Japan) GC/MS QP-2010 Ultra system equipped with an AOC-5000 autosampler was used for the automated analyses. Before use, SPME fibers were conditioned in accordance with the manufacturer’s recommendations and for each fiber blank desorption was performed. 500 µL plasma or CSF was placed in a 10 mL magnetic cap headspace vial with silicone/PTFE septum and 0.10 g sodium chloride was added. The PDMS/DVB fiber was then exposed to the headspace at 70 °C for 40 min while agitating. After this, the fiber was withdrawn into the needle and desorbed at 250 °C for 3 min into the GC injection port.

Chromatographic separations were carried out on a Restek Rxi-5MS (30 m × 0.25 mm, 0.25 µm film thickness) capillary column (Bellefonte, PA, USA) using high-purity helium as the carrier gas at a flow rate of 1.2 mL/min. The temperature program was as follows: 80 °C (hold 3 min), 20 °C/min to 120 °C, 10 °C/min to 140 °C, 20 °C/min to 180 °C, 10 °C/min to 210 °C, then 30 °C/min to 260 °C (hold 5 min). The injection temperature was set at 250 °C, and the split mode (split ratio 5:1) was used for the analysis of plasma and CSF from the AR EtOAc extract group whereas the splitless mode was used for that from the AR powder group. The spectrometers were operated in the electron-impact (EI) mode with electron energy of 70 eV and source temperature of 200 °C. The scan range was 45–450 amu, and the scan rate was 0.30 s per scan. The interface temperature was 260 °C.

Identification of the absorbed constituents was based on comparison of their mass spectral data with those from NIST 08 library and authentic standards, along with their retention index (RI) calculated using the *n*-alkanes mixture [12].

### 3.5. HPLC-APCI-IT-TOF-MS^n^ Analysis

The HPLC-APCI-IT-TOF-MS^n^ analyses were performed on a Shimadzu LCMS-IT-TOF system comprised of two LC-20AD pumps, an SIL-20AC autosampler, a CTO-20A column oven, an SPD-M20A PDA detector, a CBM-20A system controller, an APCI ion source and an IT-TOF mass spectrometer.

For pretreatment of samples, 5 mL plasma or 2.5 mL CSF was added with 10 mL or 5 mL EtOAc respectively, and then vortexed for 2 min. The mixtures were centrifuged at 3000 rpm for 15 min, and the upper organic layer was transferred to a clean tube. The mixture was again extracted with the same volume of EtOAc followed by vortex mixing and centrifugation as described above. The upper layer from the two extractions were combined and evaporated to dryness at room temperature by nitrogen. The residue was then dissolved in 100 µL methanol and 5 µL was injected into the HPLC.

Chromatographic separations were achieved at 30 °C with a Phenomenex Gemini-NX C_18_ column (5 μm particle size, 250 × 4.6 mm), using water-formic acid (100:0.1, *v/v*) (A) and acetonitrile (B) as the mobile phase. The mobile phase was delivered at a rate of 1.0 mL/min and the gradient program was as follows: 11%–42% B at 0–30 min, 42%–45% B at 30–45 min, 45%–54% B at 45–48 min, 54%–58% B at 48–55 min, 58%–60% B at 55–60 min, 60%–90% B at 60–65 min and 90%–100% B at 65–75 min. After the elution, 100% B was kept for 10 min to flush the column. For mass detection, the mass spectrometer was programmed to carry out a full scan over *m/z* 100–1000 (MS^1^) and *m/z* 50–1000 (MS^2^ and MS^3^) in positive ion detection mode. Nitrogen was used as the nebulizing gas and the flow rate was 2.0 L/min. The interface temperature, the curved desolvation line temperature and the heat block temperature was 400 °C, 250 °C and 200 °C, respectively. The detector voltage was 1.70 kV, and the drying gas pressure was 40.0 kPa. The data were processed by Shimadzu software (LCMSsolution Version 3.60, Formula Predictor Version 1.2 and AccurateMass Calculator).

## 4. Conclusions

In this paper, HS-SPME-GC-MS and HPLC-APCI-IT-TOF-MS^n^ were used for the first time to identify the volatile and non-volatile absorbed constituents in rabbits after intranasal administration of AR EtOAc extract and AR powder. For the first time, 46 absorbed constituents of AR containing 13 monoterpenes, 10 phenylpropanoids, four benzene derivatives, two alkanes, nine *N*-alkylamides and eight lignans were found could be absorbed into the rabbit plasma, whereas 28 absorbed constituents containing 14 monoterpenes, five phenylpropanoids, four benzene derivatives, two alkanes, two *N*-alkylamides and one lignan could be absorbed into the CSF. Among these absorbed constituents, 46 constituents were detected in the plasma and 28 constituents were found in the CSF after intranasal administration of AR EtOAc extract (Table 4), and 10 constituents were identified in the plasma and 6 constituents were detected in the CSF after intranasal administration of AR powder. These indicated that many types of constituents of TCM can be simultaneously absorbed at the nasal cavity into both rabbit blood and CSF. Through bibliography retrieval, the bioactivities of 33 absorbed constituents were found to be related to the pharmacological actions of AR (detailed information are listed in Table 5 and Supplemental Materials Table S1).

Furthermore, we have done several pharmacological experiments on AR EtOAc extract and its absorbed constituents. We find that AR EtOAc extract intranasally administrated exerts its analgesic effect more quickly and more potent than that of oral administration, and the four major absorbed constituents of AR (3,5-dimethoxytoluene, 2,3,5-trimethoxytoluene, 3,4,5-trimethoxytoluene, methyleugenol) and their combinations have significant analgesic effect and anti-inflammatory effect. We are applying for a Chinese patent based on these findings and for this reason the pharmacological results will be published elsewhere in the future.

These findings will enhance our understanding of the effective substances of AR, and this paper can be regarded as a paradigm for the systematic screening and identification of absorbed constituents of TCMs used in nasal therapy. Based on our results and literature retrieval, we make a hypothesis on the mechanism of additive effect of multiple constituents of TCMs, which is very worthy of further study and verification in the future.

## Supplementary Materials

Supplementary materials can be accessed at: http://www.mdpi.com/1420-3049/19/4/4857/s1.
