# Supplementary material for: Identification of Absorbed Constituents in the Rabbit Plasma and Cerebrospinal Fluid after Intranasal Administration of Asari Radix et Rhizoma by HS-SPME-GC-MS and HPLC-APCI-IT-TOF-MSn
Source: Molecules. 2014 Apr 17;19(4):4857–79. doi: 10.3390/molecules19044857 (PMC6271262; doi:10.3390/molecules19044857)
Supplement: Supplementary file 1 [file molecules-19-04857-s001.pdf]

Supplementary Materials

Table S1. Number of bioactivities of the 33 absorbed constituents related to the pharmacological effects of Asari Radix et Rhizoma.

**No.**	**Constituents**	**No. of Activities**	**Activities**
G1	α-pinene	4	Antitussive [1], anti-inflammatory [2], analgesic [3], hypotensive [4]
G2	sabinene	1	anti-inflammatory [5]
G3	β-pinene	4	anti-inflammatory [2], analgesic [6], hypotensive [4], antispasmodic [7]
G4	myrcene	4	anti-inflammatory [8], sedative [9], analgesic [10], anticonvulsant [11]
G5	α-phellandrene	3	Expectorant [12], analgesic [13], antispasmodic [7]
G6	3-carene	1	anti-inflammatory [14]
G7	limonene	7	Analgesic [15], sedative [9], anti-inflammatory [8], hypotensive [16], anti-allergic [17], anticonvulsant [11], hypothermic [16]
G8	eucalyptol	6	anti-inflammatory [18], hypotensive [19], sedative [20], analgesic [21], antispasmodic [22], antitussive [23]
G9	terpinolene	2	Antispasmodic [7], prevents oxidation of LDL (protection from atherosclerosis) [24]
G10	camphor	5	Antispasmodic [25], analgesic [26], anti-orthostatic hypotensive [27], antitussive [23], anti-inflammatory [28]
G11	eucarvone	1	anti-inflammatory [29]
G12	*l*-borneol	5	Antispasmodic [30], anti-inflammatory [31], analgesic [31], vasodilative [30], anti-allergic [32]
G13	terpinen-4-ol	4	anti-inflammatory [33], antihypertensive [34], antitussive[1], anticonvulsant [35]
G15	α-terpineol	5	Analgesic [36], anti-inflammatory [37], hypotensive [38], anticonvulsant [39], antispasmodic [22]
G16	estragole	4	Analgesic [40], anti-inflammatory [41], antispasmodic [42], hypotensive [43]
G17	3,5-dimethoxytoluene	1	Sedative [44]
G20(L6)	3,4,5-trimethoxytoluene	1	anti-inflammatory [45]
G21	methyleugenol	8	Analgesic [46], hypotensive [47], hypothermic [48], anti-allergic [49], anti-inflammatory [50], anticonvulsant [51], sedative [48], antispasmodic [52]
G26	elemicin	3	antiplatelet [53], anti-allergic [49], anti-inflammatory [49]
L4	3,4-dimethoxybenzenepropionic acid	1	Analgesic [54]

Table S1. *Cont.*

No.	Constituents	No. of Activities	Activities
L5	epipinoresinol	1	anti-inflammatory [55]
L8	(1*R*,2*S*,5*R*,6*R*)-5′-*O*-methylpluviatilol	1	anti-inflammatory [56]
L10	xanthoxylol	1	anti-allergic [49]
L11	kakuol	3	Antitussive [57], hypolipidemia [58], anti-inflammatory [45]
L12	hydroferulic acid	2	anti-inflammatory [59], antiplatelet [60]
L13	spilanthol	2	anti-inflammatory [61], analgesic [62]
L14	*l*-sesamin	2	anti-allergic [49], anti-inflammatory [56]
L15	*l*-asarinin	2	anti-inflammatory [56], anti-allergic [49]
L16	*N*-isobutyl-2*E*,4*E*,8*Z*,10*Z*-dodecatetraenamide	2	anti-inflammatory [56], antitussive [63]
L17	*N*-isobutyl-2*E*,4*E*,8*Z*,10*E*-dodecatetraenamide	4	anti-inflammatory [56], anti-allergic [49], antitussive [63], analgesic [64]
L20	*N*-isobutyl-2,4,8-dodecatrienamide	1	anti-inflammatory [65]
L21	*N*-isobutyl-2,4,8,10,12-tetradecapentaenamide	1	Antitussive [63]
L23	*N*-isobutyl-2,4-dodecadienamide	1	anti-inflammatory [65]

References

1. Saitoh, A.; Morita, K.; Ueno, K.; Yamaki, Y.; Takizawa, T.; Tokunaga, T.; Kamei, J. Effects of rosemary, plantago, and tea tree oil on the capsaicin-induced coughs in guinea pigs. *Nippon Nogeikagaku Kaishi* **2003**, *77*, 1242–1245.
2. Lorente, I.; Ocete, M.A.; Zarzuelo, A.; Cabo, M.M.; Jimenez, J. Bioactivity of the essential oil of *Bupleurum fruticosum*. *J. Nat. Prod.* **1989**, *52*, 267–272.
3. Him, A.; Ozbek, H.; Turel, I.; Oner, A.C. Antinociceptive activity of alpha-pinene and fenchone. *Pharmacol. Online* **2008**, *3*, 363–369.
4. Menezes, I.A.; Barreto, C.M.; Antoniolli, A.R.; Santos, M.R.; de Sousa, D.P. Hypotensive activity of terpenes found in essential oils. *Z. Naturforsch.* **2010**, *65*, 562–566.
5. Yao, Q.S.; Chiou, G.C.Y. Inhibition of crystallins-induced inflammation in rabbit eyes with five phytogenic compounds. *Zhongguo Yaoli Xuebao* **1993**, *14*, 13–17.
6. Liapi, C.; Anifandis, G.; Chinou, I.; Kourounakis, A.P.; Theodosopoulos, S.; Galanopoulou, P. Antinociceptive properties of 1,8-cineole and β-pinene, from the essential oil of *Eucalyptus camaldulensis* leaves, in rodents. *Planta Med.* **2007**, *73*, 1247–1254.
7. Riyazi, A.; Hensel, A.; Bauer, K.; Geissler, N.; Schaaf, S.; Verspohl, E.J. The effect of the volatile oil from ginger rhizomes (*Zingiber officinale*), its fractions and isolated compounds on the 5-HT3 receptor complex and the serotoninergic system of the rat ileum. *Planta Med.* **2007**, *73*, 355–362.
8. Souza, M.C.; Souza, M.C.; Siani, A.C.; Ramos, M.F.; Menezes-de-Lima, O.J.; Henriques, M.G. Evaluation of anti-inflammatory activity of essential oils from two Asteraceae species. *Pharmazie* **2003**, *58*, 582–586.
9. Do, V.T.; Furtado, E.C.; Santos, J.G.; Viana, G.S. Central effects of citral, myrcene and limonene, constituents of essential oil chemotypes from *Lippia alba* (Mill.) NE Brown. *Phytomedicine* **2002**, *9*, 709–714.
10. Rao, V.S.; Menezes, A.M.; Viana, G.S. Effect of myrcene on nociception in mice. *J. Pharm. Pharmacol.* **1990**, *42*, 877–878.
11. Viana, G.S.; do Vale, T.G.; Silva, C.M.; Matos, F.J. Anticonvulsant activity of essential oils and active principles from chemotypes of *Lippia alba* (Mill.) NE Brown. *Biol. Pharm. Bull.* **2000**, *23*, 1314–1317.
12. Lu, L.X.; Li, M.; Zhao, L.; Lu, Q. Sources, synthesis and application of phellandrene. *J. Anhui Agric. Sci.* **2010**, *38*, 14361–14363.
13. Lima, D.F.; Brandão, M.S.; Moura, J.B.; Leitão, J.M.; Carvalho, F.A.; Miúra, L.M.; Leite, J.R.; Sousa, D.P.; Almeida, F.R. Antinociceptive activity of the monoterpene alpha-phellandrene in rodents: Possible mechanisms of action. *J. Pharm. Pharm.* **2012**, *64*, 283–92.
14. Ocete, M.A.; Risco, S.; Zarzuelo, A.; Jimenez, J. Pharmacological activity of the essential oil of *Bupleurum gibraltaricum*: Anti-inflammatory activity and effects on isolated rat uteri. *J. Ethnopharm.* **1989**, *25*, 305–313.
15. Do, A.J.; Silva, M.I.; Neto, M.R.; Neto, P.F.; Moura, B.A.; de Melo, C.T.; de Araújo, F.L.; de Sousa, D.P.; de Vasconcelos, P.F.; de Vasconcelos, S.M.; *et al.* Antinociceptive effect of the monoterpene R-(+)-limonene in mice. *Biol. Pharm. Bull.* **2007**, *30*, 1217–20.

16. Tsuji, M.; Fujisaka, Y.; Yamachika, K.; Nakagami, K.; Fujisaki, F.; Mito, M.; Aoki, T.; Kinoshita, S.; Okubo, A.; Watanabe, I. Studies on d-limonene, as gallstone solubilizer. I. general pharmacological studies. *Oyo Yakuri* **1974**, *8*, 1439–1459.
17. Cariddi, L.; Escobar, F.; Moser, M.; Panero, A.; Alaniz, F.; Zygadlo, J.; Sabini, L.; Maldonado, A. Monoterpenes isolated from *Minthostachys verticillata* (Griseb.) epling essential oil modulates immediate-type hypersensitivity responses *in vitro* and *in vivo*. *Planta Med.* **2011**, *77*, 1687–1694.
18. Juergens, U.R.; Dethlefsen, U.; Steinkamp, G.; Gillissen, A.; Repges, R.; Vetter, H. Anti-inflammatory activity of 1.8-cineol (eucalyptol) in bronchial asthma: A double-blind placebo-controlled trial. *Respir. Med.* **2003**, *97*, 250–256.
19. Soares, M.C.; Damiani, C.E.; Moreira, C.M.; Stefanon, I.; Vassallo, D.V. Eucalyptol, an essential oil, reduces contractile activity in rat cardiac muscle. *Braz. J. Med. Biol. Res.* **2005**, *38*, 453–461.
20. Ortiz, D.U.A.; Martín, M.L.; Montero, M.J.; Morán, A.; San, R.L. Sedating and antipyretic activity of the essential oil of *Calamintha sylvatica* subsp. *ascendens*. *J. Ethnopharm.* **1989**, *25*, 165–171.
21. Santos, F.A.; Rao, V. Antiinflammatory and antinociceptive effects of 1, 8-cineole a terpenoid oxide present in many plant essential oils. *Phytother. Res.* **2000**, *14*, 240–244.
22. Ponce-Monter, H.; Campos, M.G.; Pérez, S.; Pérez, C.; Zavala, M.; Macías, A.; Oropeza, M.; Cárdenas, N. Chemical composition and antispasmodic effect of *Casimiroa pringlei* essential oil on rat uterus. *Fitoterapia* **2008**, *79*, 446–450.
23. Kumar, N.; Nepali, K.; Sapra, S.; Bijjem, K.; Reddy, V.; Kumar, R.; Suri, O.P.; Dhar, K.L. Effect of nitrogen insertion on the antitussive properties of menthol and camphor. *Med. Chem. Res.* **2012**, *21*, 531–537.
24. Grassmann, J.; Hippeli, S.; Spitzenberger, R.; Elstner, E.F. The monoterpene terpinolene from the oil of *Pinus mugo* L. in concert with α-tocopherol and β-carotene effectively prevents oxidation of LDL. *Phytomedicine* **2005**, *12*, 416–423.
25. Astudillo, A.; Hong, E.; Bye, R.; Navarrete, A. Antispasmodic activity of extracts and compounds of *Acalypha phleoides* Cav. *Phytother. Res.* **2004**, *18*, 102–106.
26. Xu, H.; Blair, N.T.; Clapham, D.E. Camphor activates and strongly desensitizes the transient receptor potential vanilloid subtype 1 channel in a vanilloid-independent mechanism. *J. Neurosci.* **2005**, *25*, 8924–8937.
27. Georg Beltz, G.; Loew, D. Dose-response related efficacy in orthostatic hypotension of a fixed combination of D-camphor and an extract from fresh crataegus berries and the contribution of the single components. *Phytomedicine* **2003**, *10*, 61–67.
28. Schwarz, J.; Weisspapir, M. Vehicle for Topical Delivery of Anti-Inflammatory Compounds. U.S. Patent 20060241175 A1, 26 October 2006.
29. Otsubo, E. Anti-inflammatory effects of volatile compound of macrophage migration inhibitory factor (MIF) induction of inflammatory in macrophage cell. *Kinki Daigaku Kogakubu Kenkyu Hokoku* **2010**, *44*, 1–7.
30. Bach, T. Preclinical and clinical overview of terpenes in the treatment of urolithiasis. *Eur. Urol. Suppl.* **2010**, *9*, 814–818.
31. Sun, X.P.; Ou, L.J.; Mi, S.Q.; Wang, N.S. Analgesic and anti-inflammation effect of borneol. *Tradit. Chin. Drug Res. Clin. Pharm.* **2007**, *5*, 8.

32. Watanabe, K.; Yano, S.; Horie, T.; Kachia, R.S.; Ikegami, F.; Yamamoto, Y.; Fujimori, H.; Kasai, M. Borneol as Allergy Inhibitor. JP 06211713, 2 August 1994.
33. Taga, I.; Lan C.Q.; Altosaar, I. Plant essential oils and mastitis disease: Their potential inhibitory effects on pro-inflammatory cytokine production in response to bacteria related inflammation. *Nat. Prod. Commun.* **2012**, *7*, 675–682.
34. Lahlou, S.; Interaminense, L.F.; Leal-Cardoso, J.H.; Duarte, G.P. Antihypertensive effects of the essential oil of *Alpinia zerumbet* and its main constituent, terpinen-4-ol, in DOCA-salt hypertensive conscious rats. *Fundam. Clin. Pharm.* **2003**, *17*, 323–330.
35. De Sousa, D.P.; Nóbrega, F.F.; de Morais, L.C.; de Almeida, R.N. Evaluation of the anticonvulsant activity of terpinen-4-ol. *Z. Naturforsch.* **2009**, *64*, 1–5.
36. Ghelardini, C.; Galeotti, N.; Mazzanti, G. Local anaesthetic activity of monoterpenes and phenylpropanes of essential oils. *Planta Med.* **2001**, *67*, 564–566.
37. Shimon, B.S. Anti-inflammatory and antimicrobial activity of diverse terpenoids found in ZufGlobus product (FlorMel). Available online http://www.nuvitalityproducts.co.il/images/skira-madait-floramel.pdf (accessed on 27 January 2014).
38. Ribeiro, T.P.; Porto, D.L.; Menezes, C.P.; Antunes, A.A.; Silva, D.F.; de Sousa, D.P.; Nakao, L.S.; Braga, V.A.; Medeiros, I.A. Unravelling the cardiovascular effects induced by α-terpineol: A role for the nitric oxide-cGMP pathway. *Clin. Exp. Pharm. Phys.* **2010**, *37*, 811–816.
39. De Sousa, D.P.; Quintans, L., Jr; de Almeida, R.N. Evolution of the anticonvulsant activity of α-terpineol. *Pharm. Biol.* **2007**, *45*, 69–70.
40. Leal-Cardoso, J.H.; Matos-Brito, B.G.; Lopes-Junior, J.E.; Viana-Cardoso, K.V.; Sampaio-Freitas, A.B.; Brasil, R.O.; Coelho-De-Souza, A.N.; Albuquerque, A.A. Effects of estragole on the compound action potential of the rat sciatic nerve. *Braz. J. Med. Biol. Res.* **2004**, *37*, 1193–1198.
41. Ponte, E.L.; Sousa, P.L.; Rocha, M.V.; Soares, P.M.; Coelho-de-Souza, A.N.; Leal-Cardoso, J.H.; Assreuy, A.M. Comparative study of anti-edematogenic effects of anethole and estragole. *Pharm. Rep.* **2012**, *64*, 984–990.
42. Coelho De Souza, A.N.; Lahlou, S.; Barreto, J.E.; Yum, M.E.; Oliveira, A.C.; Oliveira, H.D.; Celedônio, N.R.; Feitosa, R.G.; Duarte, G.P.; Santos, C.F.; *et al.* Effects of the essential oil of *Croton zehntneri*, and its constituent estragole on intestinal smooth muscle. *Phytother. Res.* **1997**, *11*, 299–304.
43. de Siqueira, R.J.B.; Magalhães, P.J.; Leal-Cardoso, J.H.; Duarte, G.P.; Lahlou, S. Cardiovascular effects of the essential oil of *Croton zehntneri* leaves and its main constituents, anethole and estragole, in normotensive conscious rats. *Life Sci.* **2006**, *78*, 2365–2372.
44. Okazaki, Y.; Okazaki, Y.; Takashima,Y.; Nakamura, S.; Yomogida, K.; Tanida, M. Sedative effect-providing fragrance modifier. Available online: http://www.google.com.mx/patents/US7968606 (accessed on 27 January 2014).
45. Han, A.R.; Kim, H.J.; Shin, M.; Hong, M.; Kim, Y.S.; Bae, H. Constituents of *Asarum sieboldii* with inhibitory activity on lipopolysaccharide (LPS)-induced NO production in BV-2 microglial cells. *Chem. Biodivers.* **2008**, *5*, 346–351.
46. Yano, S.; Suzuki, Y.; Yuzurihara, M.; Kase, Y.; Takeda, S.; Watanabe, S.; Aburada, M.; Miyamoto, K. Antinociceptive effect of methyleugenol on formalin-induced hyperalgesia in mice. *Eur. J. Pharmacol.* **2006**, *553*, 99–103.

47. Lahlou, S.; Figueiredo, A.F.; Magalhães, P.J.; Leal-Cardoso, J.H.; Gloria, P.D. Cardiovascular effects of methyleugenol, a natural constituent of many plant essential oils, in normotensive rats. *Life Sci.* **2004**, *74*, 2401–2412.
48. Jiang, Y.; Liu, G.Q.; Ma, J.R.; Xie, L.; Wu, H.Q. The pharmacological studies on methyl-eugenol. *Yao Xue Xue Bao* **1982**, *17*, 87–92.
49. Hashimoto, K.; Yanagisawa, T.; Okui, Y.; Ikeya, Y.; Maruno, M.; Fujita, T. Studies on anti-allergic components in the roots of *Asiasarum sieboldi*. *Planta Med.* **1994**, *60*, 124–127.
50. Choi, Y.K.; Cho, G.S.; Hwang, S.; Kim, B.W.; Lim, J.H.; Lee, J.C.; Kim, H.C.; Kim, W.K.; Kim, Y.S. Methyleugenol reduces cerebral ischemic injury by suppression of oxidative injury and inflammation. *Free Radic. Res.* **2010**, *44*, 925–935.
51. Sayyah, M.; Valizadeh, J.; Kamalinejad, M. Anticonvulsant activity of the leaf essential oil of *Laurus nobilis* against pentylenetetrazole-and maximal electroshock-induced seizures. *Phytomedicine* **2002**, *9*, 212–216.
52. Lima, C.C.; Criddle, D.N.; Coelho-de-Souza, A.N.; Monte, F.J.; Jaffar, M.; Leal-Cardoso, J.H. Relaxant and antispasmodic actions of methyleugenol on guinea-pig isolated ileum. *Planta Med.* **2000**, *66*, 408–411.
53. Grice, I.D.; Rogers, K.L.; Griffiths, L.R. Isolation of bioactive compounds that relate to the anti-platelet activity of *Cymbopogon ambiguus*. *Evid. Based Complement. Alternat. Med.* **2011**, *2011*, doi:10.1093/ecam/nep213.
54. He, Q.; Lu, Y.Q.; Cai, D.Q.; Cui, Y.J.; Lai, J.; Wang, H.S. Analgesic principles from Maoju (*Piper arboricola*). *Zhongcaoyao* **1981**, *12*, 433–435.
55. Lu, C.H.; Li, Y.Y.; Li, L.J.; Liang, L.Y.; Shen, Y.M. Anti-inflammatory activities of fractions from Geranium nepalense and related polyphenols. *Drug Discov. Ther.* **2012**, *6*, 194–197.
56. Quang, T.H.; Ngan, N.T.; Minh, C.V.; Kiem, P.V.; Tai, B.H.; Thao, N.P.; Song, S.B.; Kim, Y.H. Anti-inflammatory and PPAR transactivational effects of secondary metabolites from the roots of *Asarum sieboldii*. *Bioorg. Med. Chem. Lett.* **2012**, *22*, 2527–2533.
57. Kosuge, T.; Yokota, M.; Nukaya, H.; Gotoh, Y.; Nagasawa, M. Studies on antitussive principles of Asiasari radix. *Chem. Pharm. Bull.* **1978**, *26*, 2284–2285.
58. Xiao, P.G.; Liu, C.X. Pharmacology, pharmacokinetics and toxicology of Chinese traditional medicine for stroke therapy. *Asian J. Drug Metab. Pharmacokinet.* **2005**, *5*, 83–124.
59. Larrosa, M.; Luceri, C.; Vivoli, E.; Pagliuca, C.; Lodovici, M.; Moneti, G.; Dolara, P. Polyphenol metabolites from colonic microbiota exert anti-inflammatory activity on different inflammation models. *Mol. Nutr. Food Res.* **2009**, *53*, 1044–1054.
60. Rechner, A.R.; Kroner, C. Anthocyanins and colonic metabolites of dietary polyphenols inhibit platelet function. *Thromb. Res.* **2005**, *116*, 327–334.
61. Wu, L.; Fan, N.C.; Lin, M.H.; Chu, I.R.; Huang, S.J.; Hu, C.Y.; Han, S.Y. Anti-inflammatory effect of spilanthol from *Spilanthes acmella* on murine macrophage by down-regulating LPS-induced inflammatory mediators. *J. Agric. Food Chem.* **2008**, *56*, 2341–2349.
62. Boonen, J.; Baert, B.; Roche, N.; Burvenich, C.; de Spiegeleer, B. Transdermal behaviour of the *N*-alkylamide spilanthol (affinin) from *Spilanthes acmella* (Compositae) extracts. *J. Ethnopharmacol.* **2010**, *127*, 77–84.

63. Greger, H. Alkamides: Structural relationships, distribution and biological activity. *Planta Med.* **1984**, *50*, 366–375.
64. Phrutivorapongkul, A.; Chaiwon, A.; Vejabhikul, S.; Netisingha, W.; Chansakaow, S. An anesthetic alkamide and fixed oil from *Acmella oleracea*. *J. Health Res.* **2008**, *22*, 97–99.
65. Woelkart, K.; Bauer, R. The role of alkamides as an active principle of echinacea. *Planta Med.* **2007**, *73*, 615–623.

© 2014 by the authors; licensee MDPI, Basel, Switzerland. This article is an open access article distributed under the terms and conditions of the Creative Commons Attribution license (http://creativecommons.org/licenses/by/3.0/).
